# In Vitro Digestibility Assessment of Whey from Goat and Camel Milk Fermented with *Lactobacillus helveticus* for Use as a Base in Formulating Follow-On Formula

**DOI:** 10.3390/foods13040570

**Published:** 2024-02-14

**Authors:** Noura S. M. Al-Nassir, Sally S. Sakr

**Affiliations:** 1 Department of Food Science and Human Nutrition, College of Agriculture and Veterinary Medicine, Qassim University, Buraydah 51452, Saudi Arabia; nnasar@qu.edu.sa; 2 Dairy Science Department, Faculty of Agriculture, Cairo University, Giza 12613, Egypt

**Keywords:** whey, goat milk, camel milk, *Lactobacillus helveticus*, in vitro, digestion, antioxidant activity, nutrition, food supply

## Abstract

Follow-on formulas are necessary for newborns that are unable to breastfeed. Thus, the development of formulas more tailored to infants’ needs is highly important. Recently, using camel milk, goat milk, and sweet milk whey in the formulation of follow-on formulas has gained researchers’ attention. Moreover, developing postbiotic systems to create formulas that mimic human milk, are easy to digest, improve compatibility with an infant’s gut, and boost immunity is crucial. Thus, this study aimed to create and assess different formulations using fermented whey from camel and goat milks. The fermentation process involved the use of *Lactobacillus helveticus* as a probiotic and proteolytic lactic acid bacterium strain. The study monitored the proteolytic activity and antioxidant properties of sweet whey produced from cow, camel, and goat milks during the fermentation process with *L. helveticus*. Also, three different milk fat blends were recombined using edible vegetable oils (coconut oil, rice bran oil, and canola oil) and then they were used to formulate follow-on formulas with a similar fat composition to human milk. Finally, the prepared formulas were tested for their in vitro digestibility and antioxidant activity before and after digestion. The *L. helveticus* strain had high proteolytic activity towards whey proteins from all the types of milk used in the study. A fermentation time of 6 h produced a higher proteolytic degree and antioxidant activity than 2 and 4 h of fermentation. No significant differences were observed for proteolytic degree and antioxidant activity between 6 and 12 h of fermentation for the cow, camel, and goat whey samples. Regarding the fat blends, animal milk fat, rice bran oil, and canola oil in a fat combination were essential to provide the required amount of unsaturated fatty acids in the follow-on formulas, especially the linoleic acid–α-linolenic acid (LA:ALA) ratio. Adding coconut oil in small amounts to the follow-on formulas provided the required amounts of saturated fatty acids, especially lauric and meristic acids. The follow-on formula based on cow or goat milk whey fermented with *L. helveticus* released more free amino acids (mmol tyrosine equivalent mL^−1^) with high levels of antioxidants compared to unfermented ones. The release of free amino acids in the follow-on formula based on camel milk whey was not affected by fermentation. Our results recommend using *L. helveticus* in the fermentation of follow-on formulas based on camel and goat whey instead of formulas based on cow milk proteins.

## 1. Introduction

It is well-known that the best nutritional option for the newborn is their mother’s milk. Human milk contains numerous factors that protect against atopic disorders, minimize the risk of gastrointestinal and respiratory infections, and are compatible with the infant’s digestive system, as they contain bioactive components that are important for gut microbial colonization and immune maturation. However, breastfeeding is not always possible [1,2]. In many cases, follow-on formulas become the sole option for feeding infants younger than six months. Only 39% of infants worldwide under six months of age are exclusively breastfed [3], and no more than 15% of infants may receive continuous breastfeeding along with solid foods for up to two years [4]. Hence, in those cases, there is always a solid necessity to develop suitable alternatives for the mother’s milk [5,6,7].

Cow milk-based infant and follow-on formulas have been used as substitutes for human milk. This is commonly due to its high production volume, established distribution chains, and functional components. On the other hand, bovine milk has higher protein and mineral content and less lactose than human milk. The casein content in bovine milk is eight times higher than that of human milk. Infant formula should adjust the whey-to-casein ratio of bovine milk to resemble that of mature human milk, which is 60:40 [8]. As a result, many nutritional problems were reported from its use for infant feeding, primarily due to cows’ milk protein allergies and symptoms of gastrointestinal discomfort in infants [9,10,11,12]. Thus, hydrolyzed and fermented cow milk-based infant formulas with or without live probiotic bacteria became an excellent option to address the health problems related to the feeding of babies with regular formulas [10,13].

Fermented follow-on formula is manufactured using a method distinct from the one used for regular follow-on formula. During the production process, the product undergoes a fermentation procedure to acidify it, which has the potential to enhance protein digestibility and other health benefits. The inclusion of non-digestible oligosaccharides in fermented formulas can also offer various advantages, such as reducing the duration of crying [14]. Although fermented formulas do not contain live bacteria, they may contain postbiotics, which are compounds released from food by microorganisms that promote health and well-being. Additionally, certain experimental fermented formulas include bioactive compounds produced by specific strains of bacteria that are combined with prebiotics. These differences in the production process and composition distinguish fermented follow-on formula from regular follow-on formula [15,16].

Recently, the health benefits of cheese whey have attracted both nutritionists and consumers. Thus, the valorization of this highly nutritional and immune enhancer by-product by using it as a base for infant feeding became one of the most attractive solutions that can help solve the problems related to the use of cow milk-based formulas in infant feeding [17,18]. Consequently, infant formulas can be based on the whey fraction, casein fraction, or both. Suppose both fractions are used in the infant formula. In that case, fractionation is still applied for whey and casein separation, and the ratio is adjusted later (more infant formulas are based on whey proteins compared to casein since they are more readily available due to the focus on the optimal utilization of whey after cheese production [19]). Moreover, hydrolyzed whey-based formulas are effective in reducing the risk of cow’s milk allergies in high-risk infants. These formulas are more easily digestible and can promote better gastrointestinal tolerance [20]. Consequently, developing newer infant formulas and improving existing ones by enhancing their functional properties is a task for researchers and scientists. It can be achieved by using the milk of non-bovine mammals [8].

Because of the higher similarity of camel and goat milks in composition to human milk compared to cow milk (Table 1), infant formulas have been predominantly manufactured using camel and goat milks and their proteins to provide a suitable protein source that is healthier and more easily digestible for infants [17,21,22,23]. Camel milk is distinct from the milk of other species and bears a resemblance to human milk, owing to its low levels of κ-casein and the absence of β-lactoglobulin. As a result, it stands as a promising option for individuals who suffer from a cow’s milk protein allergy [22,23,24,25]. Furthermore, camel milk is rich in essential nutrients such as vitamin C, iron, and potassium, while being low in fat and lactose content. The milk proteins in camel milk release unique bioactive peptides during gastrointestinal digestion, rendering it an excellent source of natural antioxidants. Camel whey protein, in particular, is recognized for its ability to mitigate oxidative stress, enhance immune system function, and elevate glutathione levels. It is replete with lactoferrin, lactalbumin, lactoglobulins, lactoperoxidase, lysozyme, and immunoglobulins [23,26,27]. On the same track, goat milk is deemed a beneficial alternative for infants and patients who suffer from cow milk allergies [1]. It is considered an appealing food source due to its higher nutritional value and enhanced digestibility as compared to cow milk, resulting from the formation of softer curd [21]. Goat milk has lower levels of αs1-casein, and its fat content comprises higher levels of medium-chain fatty acids and smaller fat globules than cow milk [12].

Additionally, whey from goat milk contains approximately 55% of milk nutrients, including soluble proteins, lactose, vitamins, minerals, and minimum fat content [17]. According to a study conducted by Zhou et al. [28], there is a growing demand for infant formulas containing goat milk. The study found that goat milk formulae provide identical growth and nutritional outcomes in infants as compared to a standard whey-based cow milk formula.

**Table 1:** Average composition of bovine, non-bovine, and human milk [29,30,31].

Component (%)	Bovine		Non-Bovine			Human
	Cow	Buffalo	Camel	Small Ruminants		
				Goat	Sheep	
TS	12.73	18.64	12.81	12.4	17.1	11.5
Protein	3.42	4.70	2.33	3.70	5.50	0.9
Casein:whey	82:18	82.18	73:27	78:22	76:24	27:73
Fat	4.09	8.30	4.10	3.80	5.90	3.4
Lactose	4.82	4.80	4.81	4.10	4.80	7
Ash	0.72	0.84	0.73	0.80	0.90	0.2

The present study aimed to develop and evaluate follow-on formulas that offer improved nutritional value, higher protein bioavailability, and greater tolerance than regular formulas while mimicking the fat content of human milk suitable for infants who are unable to breastfeed exclusively. The proposed method involves the fermentation of goat or camel milk whey using *L. helveticus* to produce postbiotic follow-on formulas.

## 2. Materials and Methods

### 2.1. Materials

After they provided written informed consent regarding volunteering with their milk in the study, human milk samples were collected from volunteer lactating mothers aged 25–30 years during the first six months after giving birth, and they were asked to collect samples (5 mL milk from each mother for only one time) after infant feeding. Skimmed milk powder (97% TS) was purchased from Watein-Biolife Advanced Medicals and Diagnostics, Riyadh, Saudi Arabia. Raw cow milk from Al-Zunaidi’s private farm and raw goat milk (from Cypriot goats) from Al-Nawader’s private farm were gained immediately after milking. Those farms are located in the Unaizah governorate, Qassim region, Saudi Arabia.

The *L. helveticus* (CNRZ32) strain was brought as a gift from the dairy microbiological lab, Centre National de Recherche Zootechnique, Jouy En-Josas, France. For bacterial strain activation, the De Man, Rogosa, and Sharpe (MRS) broth medium was purchased from Condalab, Calle Forja 9, 28850, Torrejón de Ardoz, Madrid, Spain.

Chemosin (FAR-M sticks, Chr. Hansen laboratories, Copenhagen, Denmark.) suitable for camel milk coagulation was purchased from Misr Food Additives (MIFAD, Badr City El Roubeky Rd., Area 250 Fadan Plot 154, Badr, Egypt). Pepsin (1:3000) was supplied from Aldon corporation (221 Rochester Street, Avon, NY 14414, USA), and Pancreatin from porcine pancreas (≥3 × USP specifications: Sigma-Aldrich, Merck Group, St. Louis, MO, USA) was purchased from Bayouni Trading Co. Ltd., Riyadh St., Cross 21 Bayouniya Alkhobar, Khobar, Riyadh, Saudi Arabia. All chemicals used in the study were of analytical grade. Chemicals for each experimental method are mentioned in detail under the method of analysis described below.

### 2.2. Study Design

**Figure 1:**
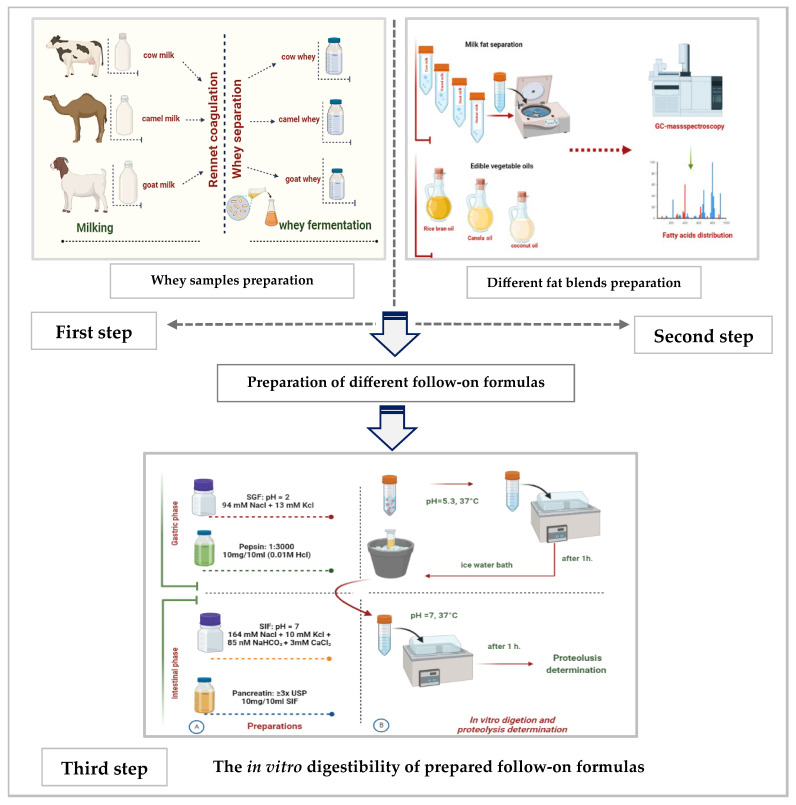
The summarization of the study’s three sequential steps (A: the preparation steps and B: the digestion steps).

This manuscript is the first output of a project that was approved by the Committee of Research Ethics, Deanship of Scientific Research, Qassim University (24-72-01). The study was planned to be conducted in in vitro and in vivo (in an animal model) studies. This paper is an in vitro study, and no patients are in our study. Only volunteer mothers provided us with their milk for analysis after they were informed that the samples would be used for scintefic research The current in vitro research study was conducted in three sequential steps, as shown in Figure 1.

During the first step, sweet whey from cow, camel, and goat milks was separated after rennet coagulation and then lyophilized. Later on, sweet whey samples from the three species were fermented separately by *L. helveticus*. Finally, all non-fermented and fermented whey samples were analyzed for their chemical composition, physicochemical characteristics, degree of proteolysis, total antioxidant activity, and hydrolysis of protein by electrophoresis.

In the second step, a mixture of fat and oil that mimics fatty acid (FA) content and type in human milk was prepared. For that, cream from human, camel, and goat milks was separated by centrifuge under cooling. Different separated milk fats and refined oils (coconut oil, rice bran oil, and canola oil) were checked for their fatty acid content and type by gas chromatography–mass spectrometry (GC-MS, Thermo Scientific Trace GC Ultra Gas Chromatograph, Waltham, MA, USA).

The third step involved using the results obtained from the first and second steps. Specifically, fermented whey samples from the first step and a chosen fat and oil mix from the second step were used to create three different follow-on formulas. These formulas were then evaluated based on their in vitro digestibility, as well as their total antioxidant activity against both a mother’s milk sample and two regular follow-on formulas available in the local market.

### 2.3. Preparation Methods

#### 2.3.1. Preparation of Whey from Different Milk Sources

Sweet whey from cow, camel, and goat milk was prepared as described by Carter et al. [32] with some modifications. Briefly, two litters from each milk type were separately warmed in a water bath until the temperature reached 37 °C. Subsequently, according to the supplier’s recommendations, the rennet enzyme (FAR-M sticks, Chr. Hansen laboratories, Copenhagen, Denmark) was added at a concentration of 0.01 g 2 L^−1^ to cow and goat milk and at a concentration of 0.03 g 2 L^−1^ to camel milk. After that, each milk type was incubated at 37 °C until complete coagulation occurred. The curd from each milk type was then left on a cheesecloth for the next day under cold conditions until the whey thoroughly drained out of the curd. Finally, different whey samples were collected. A portion of each whey was freeze-dried (CHRIST, Alpha 1–2 LD plus, Germany) for 96 h at −52 °C under a pressure of 0.032 mbar, and the rest of the whey samples were kept under freezing at −25 °C until use.

#### 2.3.2. Starter Culture Preparation and Activation

The *L. helveticus* strain was activated in MRS broth at 37 °C overnight five times, followed by a sixth passage in sterilized skim milk (9% TS containing 1% glucose) at 37 °C overnight to prepare a starter culture available to be used in the rest of the study [33]. The ability of *L. helveticus* to hydrolyze milk whey proteins over 2, 4, 6, and 12 h of incubation at 40 °C was monitored after inoculation of sweet whey prepared from each type of milk with *L. helveticus* starter culture (2%).

#### 2.3.3. Milk Fat Separation and Different Fat Blend Formulation

Fat from the cow, camel, goat, and human milks was separated under cooling (4 °C) at 5000 rpm for 10 min using a centrifuge (Hermle Labortechnik GmbH, Wehingen, Germany). The separated fat on the upper layer of the centrifugation tube for each milk type was then kept under −25 °C, and the distribution of fatty acids was analyzed by GC-mass spectroscopy (GC-information).

For the formulation of different fat blends used under the current study, both milk fat sources (cow, camel, goat, and human milk fat) and edible vegetable oils (rice bran, canola, and coconut oil) were analyzed by GC-mass spectroscopy for detecting the relative distribution of their fatty acid content. Then, three different fat blends (cow-based fat blend, camel milk fat blend, and goat milk fat blend) were prepared according to the recommendations of the European Society for Pediatric Gastroenterology, Hepatology, and Nutrition (ESPGHAN) [34] to approximately mimic the distribution of the fatty acids in human milk.

#### 2.3.4. Preparation of Different Follow-On Formulas

Depending on data collected from the first and second steps under the current research, three suggested follow-on formulas were made (cow milk whey-based follow-on formula, camel milk whey-based follow-on formula, and goat milk whey-based follow-on formula). The in vitro digestibility of the three prepared formulas was then determined. The gastric and intestinal phases of the in vitro digestibility were adopted to assess the digestibility of the different prepared infant formulas [35,36,37].

#### 2.3.5. Preparation of Soluble Nitrogen Extract (SNE) from Whey and Prepared Follow-On Formulas

To prepare the soluble nitrogen extract, the methodology proposed by Lievore et al. [38] was followed. The process involves mixing 2 mL of the sample with 1 mL of distilled water, followed by adding 5 mL of 12% (*w*/*v*) trichloroacetic acid (TCA) and mixing thoroughly. The mixture was allowed to stand for 10 min before being centrifuged at 10,000× *g* for 30 min. The supernatant was then carefully collected and frozen at a temperature of −25 °C to facilitate subsequent analyses.

### 2.4. Methods of Analysis

#### 2.4.1. Whey Yield %, Chemical Composition, pH, and Titratable Acidity (TA%)

The total solids percent (TS%) and ash % were determined according to the methods described in the Association of Official Analytical Chemists methods [39]. Fat content was analyzed using Gerber’s method [40]. The titratable acidity of milk (expressed as lactic acid %) was determined by titration with 0.1 N NaOH using phenolphthalein as an indicator. The pH of different whey samples was measured fresh, during fermentation, and at the end of fermentation by *Lactobacillus helveticus* using a digital pH meter (HANNA HI 8314 Portable).

#### 2.4.2. Proteolytic Activity

The free amino acids of different samples in the current study were determined by the OPA method, as described by Mudgil et al. [37]. Briefly, The OPA reagent was prepared from 25 mL of 100 mmol sodium tetraborate, 2.5 mL of 20% (*w*/*v*) sodium dodecyl sulfate (Sigma-Aldrich, Merck Group, St. Louis, MO, USA), 40 mg of OPA (Sigma-Aldrich, Merck Group, St. Louis, MO, USA) previously dissolved in 1 mL methanol, and 100 μL of β-mercaptoethanol (Sigma-Aldrich, Merck Group, St. Louis, MO, USA). The total volume of the mixture was increased to 50 mL by distilled water. The OPA reagent was freshly prepared and used within 1 h of preparation. For the assay, 30 μL of prepared TCA extract was poured into a 96-well microplate reader (Multiskan Sky, Thermo Fisher Scientific, Cambridge, MA, USA), and 270 μL of OPA reagent was added to each well. The absorbance was measured at 340 nm. A standard curve of tyrosine ranging from 0.039 to 10 μM was used to calculate the free amino acid content as μmol tyrosine equivalent mL^−1^ sample.

#### 2.4.3. SDS-PAGE Electrophoresis

The electrophoresis techniques were applied in the central research laboratory, female student’s campus, King Saud University, Riyadh, Saudi Arabia. Sodium dodecyl sulfate-polyacrylamide gel electrophoresis (SDS-PAGE electrophoresis) was used to measure protein degradation in samples under the current study. Samples (8 μL) were suspended in 5 μL of sample buffer [41] and heated at 99 °C for 5 min, then 30 μL of each sample was injected. The thermoscientific PageTMRuler unstained protein ladder was used as a marker (MW, 10–200 kDa, Thermo Fisher Scientific, Waltham, MA, USA). Tricine SDS-PAGE was carried out on 17% (*w*/*v*) polyacrylamide gels on vertical slab electrophoresis cell (BIO-RAD Mini PROTEIN^®®^TGXTM Precast Gels, BIO-RAD, CA, USA) for 60 min at 120 V.

#### 2.4.4. Antioxidant Activity Assay

DPPH radical scavenging activity of samples was determined according to the modification mentioned by Mudgil et al. [37]. The decolorization of DPPH free radicals after scavenging was monitored by measuring the absorbance at 517 nm after 30 min of incubation at 37 °C using a 96-well microplate reader (Multiskan Sky, Thermo Fisher Scientific, Cambridge, MA, USA). A total of 25 μL of each soluble nitrogen extract was mixed with 275 μL DPPH reagent (0.1 mmol/L in 95% methanol) in a 96-well microplate reader and left in the dark for 30 min before the measurement. The radical scavenging activity percent (DPPH radical scavenging activity of extracts %) was calculated as follows:

DPPH radical scavenging activity (%) = (A_0_ − A_s_)/A_0_ × 100 (1)

where A_0_ is the absorbance at 517 nm of blank; A_s_ is the absorbance at 517 nm of extract.

Also, a standard curve equation using Trolox was used in calculating the μmol Trolox equivalent mL^−1^ sample.

For the reducing power method, the SNE (1 mL) was mixed with 1 mL of 0.2 M phosphate buffer (pH 6.6) and 1 mL of 1% potassium ferricyanide. The mixture was incubated at 50 °C for 20 min, followed by adding 1 mL 10% TCA. An aliquot (2 mL) from the incubation mixture was mixed with 2 mL of distilled water and 0.4 mL of 0.1% ferric chloride in test tubes. After 10 min, the absorbance of the resulting solution was measured at 700 nm. A standard curve of Ascorbic acid (7.5–500 μg) was plotted, and the ordinary curve equation was used to calculate the μg Ascorbic acid equivalent mL^−1^ sample.

#### 2.4.5. Gas Chromatography–Mass Spectrometry (GC-Mass)

The GC-MS analysis of cow, camel, goat, and human milk fats, as well as rice bran, canola, and coconut oils, was carried out using gas chromatography–mass spectrometry instrument stands with the following specifications: a TRACE GC Ultra Gas Chromatographs (THERMO Scientific Corp., Waltham, MA, USA), coupled with a thermal mass spectrometer detector (ISQ Single Quadrupole Mass Spectrometer). The GC-MS system was equipped with a TR-5 MS column (30 m × 0.32 mm i.d., 0.25 μm film thickness). Analyses were carried out using helium as carrier gas at a flow rate of 1.0 mL/min and a split ratio of 1:10 using the following temperature program: 60 °C for 1 min, rising at 4.0 °C/min to 240 °C and held for 1 min. The injector and detector were held at 210 °C. Diluted samples (1:10 hexane, *v*/*v*) of 1μL of the mixtures were continuously injected. Mass spectra were obtained by electron ionization (EI) at 70 eV, using a spectral range of *m*/*z* 40–450. The identification of the chemical constituents of the essential oil was de-convoluted using AMDIS software “www.amdis.net (accessed on 2 August 2022)” by its retention indices (relative to n-alkanes C4–C22) and mass spectrum matching to authentic standards (when available), Wiley spectral library collection, and NSIT library database.

#### 2.4.6. The In Vitro Digestibility

The method described by [42], with subsequent modifications made by [37], was used to simulate infant gastric and intestinal digestion. Briefly, for simulated infant gastric digestion (SIGD), 10 mL of different prepared infant formulas was mixed with 20 mL of simulated gastric fluid (SGF; 94 mmol NaCl, 13 mmol KCl, adjusted to pH 2 using 1 mol HCl) in a 50 mL falcon tube. Next, the pH of the mixture was readjusted to 5.3 using 1 mol HCl, and 1 mL porcine pepsin (3000 U/mL) was added. Digestion was carried out with continuous shaking in a water bath maintained at 37 °C for 1 h, and the reaction was stopped by immersing tubes in an ice water bath. For intestinal digestion, the (20 mL) was mixed with 18 mL simulated intestinal fluid (SIF mixture containing: 10 mmol KCl, 85 mmol NaHCO3, 164 mmol NaCl, 3 mmol CaCl_2_) and 0.5 mL of pancreatin solution (10 mg of ≥3 × USP pancreatin/10 mL SIF without CaCl_2_). The pH was adjusted to 7, and the tubes were homogenized; then, digestion was carried out under continuous shaking in a water bath maintained at 37 °C for 1 h. Heat shock treatment was used to stop intestinal digestion by immersing tubes in an ice water bath. The digested samples were then used for a degree of proteolysis analysis.

#### 2.4.7. Statistical Analysis

Data are expressed as the mean ± standard deviation (SD). A randomized complete block design and analysis of variance of factorial methods were carried out using the SPSS program (ver. 22). Results were considered statistically significant at *p* ˂ 0.05.

## 3. Results and Discussion

### 3.1. Whey Sample Preparation and Fermentation by L. helveticus

#### 3.1.1. Whey Sample Yield, Chemical Composition, and Fermentation Behavior

**Figure 2:**
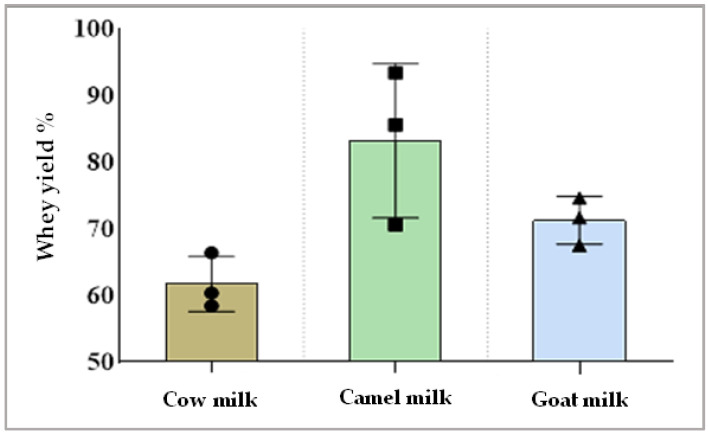
Whey gained from each type of milk after rennet coagulation (whey yield %).

Data presented in Figure 2 display the amount of whey separation obtained from different types of milk after rennet coagulation. The yield of separated whey from camel milk was higher than whey separated from cow milk or goat milk.

The chemical composition of different prepared whey (Table 2) showed that cow milk whey had higher amounts of protein and lactose than camel and goat milk whey. The percent of protein in camel milk was the lowest compared to cow or goat milk whey, while its content of ash was the highest. These findings could be related to the large amounts of whey released in camel and goat milk compared to cow milk. In other words, the smaller amounts of whey separated after rennet coagulation could be a reason for the higher concentration of whey proteins and lactose, as they were dispersed in a smaller amount of water.

#### 3.1.2. Proteolytic Degree and Antioxidant Activity of Whey Samples during Fermentation

The degree of proteolysis, using OPA methods, as well as the total antioxidant activity, using DPPH assay, were measured for different prepared whey when fresh and after 2, 4, 6, and 12 h of fermentation in order to decide the suitable and applicable fermentation period in the current study. Data gained by the OPA method, presented in Table 3, showed significant differences in proteolytic degree between all whey types at different fermentation periods. No significant differences were observed in the proteolytic degree (mmol tyrosine equivalent) in all whey types before fermentation, as they were 3.51, 3.41, and 1.57 immediately after pasteurization for cow, camel, and goat whey, respectively, while they were 3.41, 1.57, and 3.08 just after *L. helveticus* addition (Z_3_) for cow, camel, and goat whey, respectively. The highest proteolytic degrees during the fermentation period were significantly observed at 6 and 12 h of incubation for all whey samples. The calculated increase in the proteolytic degree after 12 h of fermentation was 3.68, 0.52, and 4.57 mmol tyrosine equivalent mL−1 for cow, camel, and goat whey, respectively. This finding indicated that whey proteins from cow and goat milk are more affected by the action of L. helveticus than camel milk under the conditions of the current study. Although all three types of whey gave the highest rate of protein degradation at 12 h of fermentation, the significant difference was slight between 6 and 12 h of fermentation with *L. helveticus*. A period of 6 h of fermentation was then chosen as the total fermentation period for the rest of the current study.

**Figure 3:**
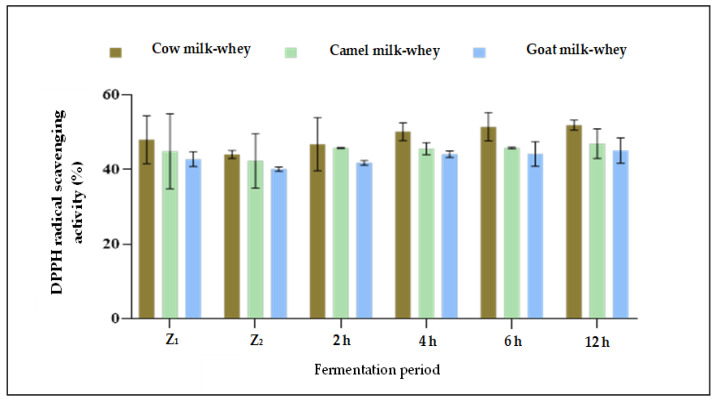
DPPH radical scavenging activity (%) of non-fermented and fermented whey samples during 12 h of fermentation with *L. helveticus*. Z_1_: Fresh whey, Z_2_: Whey immediately after pasteurization.

Regarding the DPPH radical scavenging activity, data presented in Table 4 and Figure 2 revealed that the more the time of fermentation by *L. helveticus* increased, the more the DPPH radical scavenging activity increased for all whey types. The higher radical scavenging activity percent in all fermentation times was for cow milk whey (after 2 h:1.01, after 4 h:1.08, and after 6 h:1.17 μmol Trolox equivalent mL^−1^) compared to camel and goat milk whey. No significant difference (*p* > 0.05) was detected between 6 and 12 h of fermentation. No apparent differences in the DPPH radical scavenging activity were observed between camel and goat whey samples in all fermentation times under the current study. For all three whey samples, no significant differences (*p* > 0.05) were detected between 6 and 12 h of fermentation by *L. helveticus*. Although there was an apparent increase in the proteolytic degree as a result of fermentation between 6 and 12 h for all whey samples (Table 3), there were no differences in the total antioxidant activity between the exact times of fermentation (Figure 3 and Table 4). According to the suggestion of [43], the sequences of amino acids released from native proteins in the aqueous medium could act directly as primary free radical scavengers, which may be an effect of the formation of other compounds (e.g., oligopeptides, peptides, and organic acids) in the hydrolyzed samples during fermentation. Also, they found that whey protein isolates neutralized free radicals effectively and found a significant free radical inhibition by *L. helveticus* hydrolysate, and this effect was strain-dependent.

#### 3.1.3. SDS-PAGE Electrophoretic Assay of Whey Samples during Fermentation

**Figure 4:**
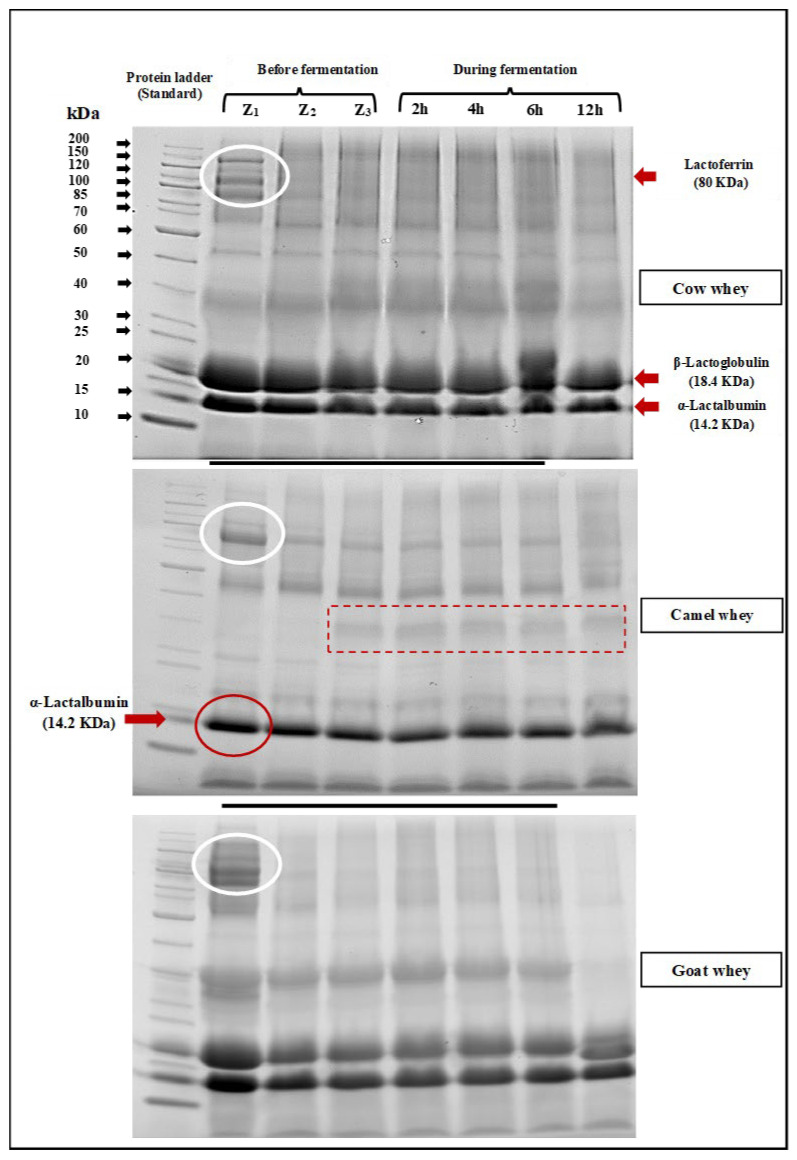
SDS-PAGE electrophoretic pattern of whey from different milk types before fermentation (Z_1_: fresh whey, Z_2_: whey immediately after pasteurization, and Z_3_: after *L. helveticus* inoculation) and during fermentation with *L. helveticus* for 12 h, where the white oval shape represents the Lactofferin band (80KDa), the red oval shape represents the α-lactalbumin band (12.2KDa), and the dashed box represents no bands for β-lactoglobulin observed in camel milk whey.

SDS-PAGE electrophoretic patterns of cow, camel, and goat milk wheys fermented by *L. helveticus* after 2, 4, 6, and 12 h of incubation at 40°C are shown in Figure 4. It is clearly observed that bands with a molecular weight of about 18 KDa, which refers to β-lactoglobulin, are not observed in camel milk whey. Bands referring to immunoglobulins, lactoferrin, and heavy chains were also observed (red rectangle). The same observation was obtained by Abdallah et al. [44]. They said that camel whey samples lack casein and contain high concentrations of IgG immunoglobulins in addition to moderate concentrations of albumin and deficient concentrations of lactoferrin. In the electrophoretic patterns of cow and goat milk wheys, they have contained bands with both α-Lactalbumin (14.2 KDa) and β-Lactoglobulin (18.3 KDa). Lactoferrin bands (80 KDa) are found in all milk whey types (Figure 4). Generally, the effect of *L. helveticus* in hydrolyzed whey protein in all whey types is clearly observed as the density of the bands gradually became fine with increasing fermentation period. Also, the bands of heavy chain (MW: 70–200 KDa) particles also became finer in density as the fermentation time increased, which means the hydrolysis with *L. helveticus* is time-dependent. The health effect of hydrolyzation of milk proteins with *L. helveticus* was reviewed by García-Burgos et al. [45], and the general conclusion was that the milk protein fermentation by this strain led to a liberation of bioactive peptides, which have been proposed as an alternative for the control of bacterial infection due to its antimicrobial and immunostimulant properties. They also suggested that infant formula should be improved by fermentation, which promotes intestinal microbiota like Bifidobacteria.

### 3.2. Preparation of Suggested Fat and Oil Blends That Mimic FA Content and Type in Human Milk

Milk fat (MF) separated from human, cow, camel, and goat milk, as well as vegetable oils (coconut oil, rice bran oil, and canola oil), were analyzed by GC-mass spectroscopy for their FA relative distribution. Data presented in Table 5 show the relative distribution of the abundant FAs identified under the conditions of the current study.

The total amount of saturated fatty acids (SFAs) was close in human milk (72.11%) to that in bovine milk fat, and ranged from 64.62% in camel milk fat to 75.42% in goat milk fat, and the dominant one was palmitic acid (C16:0). In parallel to our results regarding butyric acid (C:4), it was mentioned that studies are not able to detect butyrate in human milk; however, some studies do report the presence of butyric acid in low concentrations [46]. The unsaturated fatty acids (USFAs) were higher in camel milk fat (35.38%) than other bovines (cow milk fat: 12.93% and goat milk fat: 18.26%) or human milk fat (27.90%), and the dominant unsaturated fatty acid was oleic acid (C18:1). Our result was in agreement with Teng et al. [47], who reported that human milk is higher in USFAs than different bovine milk types, which is beneficial for infant health, since polyunsaturated fatty acid (PUSFA)-rich diets appear to reduce fatal coronary heart disease. Also, when comparing camel milk fat with cow, goat, and human milk fat, it can be observed that camel milk fat had little content of short- and medium-chain fatty acids (C2:0–C10:0), while the intermediate- and long-chain fatty acids (C:12–C:18:0) were found in higher amounts, in contrast to other milk fat types. The short- and medium-chain FAs were also found in higher amounts in human milk and goat milk than in camel milk fat, as previously mentioned [46,47].

Generally, it is well-known that milk fat triacylglycerol is composed of more than 400 different FAs, making it the most complex natural fat, and that milk lipids are not a fixed constant but vary according to many factors, including their amount and composition [48].

In parallel with results reported by Abdel-Ghany et al. [49], rice bran oil exhibited a higher amount of USFAs (53.47%) than SFAs (46.45%), where palmitic acid was dominant among all saturated fatty acids. Lauric acid was found in high amounts among fatty acids in coconut oil (25.29%). Besides lauric acid and other SFAs, myristic (28.25%), palmitic, and stearic acids were also found. Oleic acid was found to be the predominant USFA in coconut oil (4.28%). This finding was in agreement with Kostik et al. [50]. Regarding canola oil, the USFAs were higher than SFAs, and oleic acid was the dominant one (74.82%).

Depending on the data presented in Table 5 for fatty acid type and content in each milk fat type and edible vegetable oils, the different milk fat blends containing edible vegetable oils presented in Table 6 were prepared and emulsified by adding lecithin to be used in the recombination of different follow-on formulas in the third part of the current study. Also, the relative distribution of fatty acids in the formulated fat blends was determined by GC-mass spectroscopy (Table 7).

The total saturated fatty acids were 38.15%, 38.51%, and 41.86% for cow milk fat-, camel milk fat-, and goat milk fat-based blends, respectively. Different sources of milk (cow, camel, and goat) have varying levels of MUSFAs and PUSFAs. The blend of cow milk fat has 38.56% MUSFAs and 22.39% PUSFAs, camel milk fat blend has 39.49% MUSFAs and 21.16% PUSFAs, and goat milk fat blend has 53.74% MUSFAs and 21.76% PUSFAs. The linoleic acid (LA) represented about 17.95%, 17.13, and 17.46% for cow milk fat blend, camel milk fat blend, and goat milk fat blend, respectively, and the α-Linolenic acid was 3.43%, 3.09%, and 3.35% for cow milk fat blend, camel milk fat blend, and goat milk fat blend, respectively. Thus, the ratio between LA and ALA for the three blends was close to 5–15:1. Also, the sums of lauric acid and myristic acid were 9.75%, 6.265%, and 11.466%, and the trans fatty acids were 1.00%, 0.84%, and 0.64% for cow milk fat blend, camel milk fat blend, and goat milk fat blend, respectively. These results were in agreement with the recommendations of the Coordinated International Expert Group of the European Society for Pediatric Gastroenterology, Hepatology, and Nutrition (ESPGHAN) regarding the standards for the composition of infant formula [34]. They recommended that the average of saturated fatty acids and monounsaturated fatty acids be between 34 and 47% and 31 and 43%, respectively, and the trans fatty acids should not exceed 3%. Also, the sum of lauric acid and myristic acid should not exceed 20% of total FAs to avoid their potential adverse effects on cholesterol and lipoprotein levels.

### 3.3. Formulation of Different Fermented Whey-Based Follow-On Formulas and Their Properties

#### 3.3.1. Formulation of Different Fermented Whey-Based Follow-On Formulas

Six commercial follow-on formulas available in the local market were used as a reference to establish the typical chemical composition of follow-on formulas that are closer to human milk in terms of major components. The collected data revealed that the protein percentage ranged between 1.51% and 2.33%, the total fat percentage was about 3% in all documented formulas, and the lactose percentage was about 7% in most formulas. Based on the collected information and the recommendations of ESPGHAN [34], additional components were calculated and mixed to achieve the required percentages of each component in the different whey-based follow-on formulas. Additional components (g 100 mL^−1^) were added to prepare the different formulas, as shown in Table 8. The data presented in Table 7 show a change in the percentage of protein in the prepared follow-on formulas. The percentage of protein in the follow-on formula based on cow milk whey was 2.9%, while it was within 2% in both the formula based on camel milk whey and te formula based on goat milk whey. This is due to the lower absorption and weaker ability to digest and absorb bovine proteins in infants’ guts compared to the digestability of camel or goat milk proteins [51]. The whey–casein ratio achieved was 60:40, as in mature milk [52], by adding casein powder and freeze-dried whey. The percentage of fat was fixed within the range of 4% of the difference in the contents of different edible vegetable oils or the mixture of fats used in each of the prepared formulations. Also, the percentage of lactose was constant in the three formulations and constituted 7%. Lecithin was added (0.5%) as a support material for emulsifying the fat in the water emulsion.

#### 3.3.2. Proteolytic Degree and Antioxidant Activity of Different Prepared Fermented and Non-Fermented Follow-On Formulas before and after In Vitro Digestibility

##### SDS-PAGE Electrophoretic Patterns of Different Prepared Fermented and Non-Fermented Follow-On Formulas

The SDS-PAGE electrophoreses technique was applied to the three prepared follow-on formulas non-fermented and fermented for 6 h by L. helveticus before in vitro digestibility to determine the suitability of each type of fermented whey-based follow-on formulas for digestion under the simulated infant gastric and intestinal conditions.

**Figure 5:**
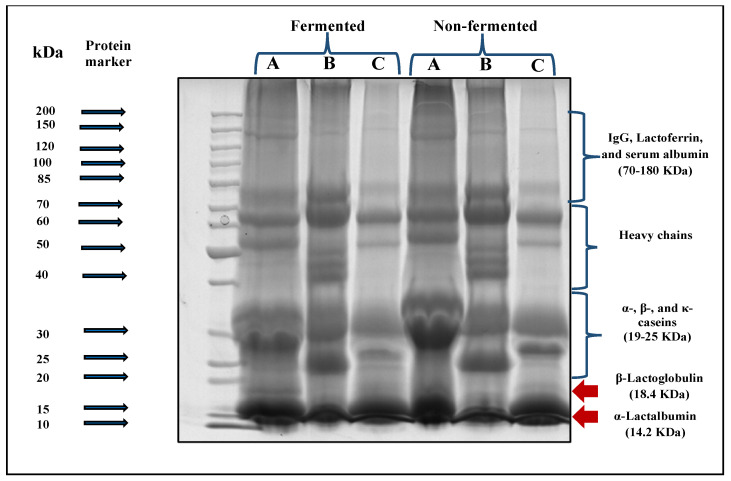
SDS-PAGE electrophoretic patterns of different prepared follow-on formulas before and after in vitro digestibility. A: Cow whey-based follow-on formula, B: camel whey-based follow- on formula, and C: goat whey-based follow-on formula.

Data presented in Figure 5 showed an evident degradation of protein fractions after fermentation for all fermented follow-on formal samples prepared under the current study. The density of β-lactoglobulin and α-lactalbumin bands (MW:14.2–18.4 KDa respectively) in cow milk whey (A)- and goat milk whey (C)-based follow-on formulas became finer bands. The same observation was also found in the casein fractions (MW: 19–25 KDa) for those formulas. Regarding the camel milk whey-based follow-on formula (B), no bands of β-lactoglobulin were found. A slighter change in the density of α-lactalbumin and casein fraction bands was observed than those of cow milk whey (A)- or goat milk whey (C)-based follow-on formulas.

##### Proteolytic Degree and Antioxidant Activity of Different Non-Fermented and Fermented Prepared Follow-On Formulas against Infant Formulas from Market and Human Milk before and after In Vitro Digestibility

Table 9 shows in vitro digestibility (OPA) and antioxidant activity (DPPH and reducing power) for three developed follow-on formulas, two commercial follow-on formulas from the local market, and a human milk sample.

The proteolytic activity, determined as absorbance at 340 nm or as mmol tyrosine equivalent mL^−1^ (Table 9), had the same trend for all samples. All prepared follow-on formulas, formulas from the local market, and human milk samples had significant (*p* ˂ 0.05) increases in the proteolytic activity when compared to the same samples before in vitro digestion except for the goat whey-based follow-on formula, which presented no significant changes (*p* > 0.05). Due to the effect of fermentation with *L. helveticus*, the proteolytic degree was significantly increased for fermented samples after the in vitro digestion compared to non-fermented ones (cow whey-based follow-on formula: 0.28 and goat whey-based follow-on formula: 0.36 mmol Tyrosine equivalent mL^−1^), except for camel whey-based follow-on formula. Moreover, the degree of proteolysis, according to the amounts in mmol tyrosine equivalent mL^−1^ before and after digestion, was increased in different amounts for all samples. Those amounts were 0.18, 0.27, 0.62, and 0.04 mmol tyrosine equivalent mL^−1^ for human milk and non-fermented cow whey-based follow-on formula, camel whey-based follow-on formula, and goat whey-based follow-on formula, respectively. For the fermented samples, the calculated amounts were 0.14, 0.19, and 0.1 for cow whey-based, camel whey-based, and goat whey-based follow-on formulas, respectively. Thus, it could be indicated that the fermentation by *L. helveticus* had a positive effect in simplifying whey proteins in cow and goat whey-based follow-on formulas. In contrast, its effect on camel milk whey was slight.

Data presented in Table 9 indicate that the antioxidant effect of all samples significantly increased (*p* < 0.05) after in vitro digestion. The release of antioxidants after digestion was the highest for the fermented goat whey-based follow-on formula (DPPH radical scavenging activity: 21.47 μmol Trolox equivalent mL^−1^), with a total DPPH radical scavenging activity of 67.60%, and fermented cow whey-based follow-on formula (DPPH radical scavenging activity: 21.35 μmol Trolox equivalent mL^−1^), with a total DPPH radical scavenging activity of 67.60%, with no significant difference between them. The antioxidant activity of non-fermented prepared follow-on formulas under the current study did not significantly (*p* > 0.05) differ from typical human milk after in vitro digestion. Among all samples, the lowest DPPH radical scavenging activity was recorded for the non-fermented cow whey-based follow-on formula (65.46%). In comparison, the camel whey-based follow-on formula became second-order, with no significant differences between non-fermented (66.70%) and fermented prepared camel whey-based follow-on formula (67.60%). As for the reducing power assay method, which did not demonstrate remarkable changes between samples, it can be said that it was not suitable for such a study. In a study carried out by El-Sayed et al. [53] to evaluate the antioxidant capacity of fermented camel milk using some single strains of *Lactobacillus sp.*, essential findings were shown regarding the antioxidant effect of fermentation by *L. helveticus*. They found that *L. helveticus* was one of the strains that had the highest scavenging activity with no relationship between the proteolysis degree and DPPH radical scavenging activity values, which indicates the importance of the type and sequence position of functional amino acids in the peptides released for this activity and that the antioxidant properties of proteins and peptides count on many factors, including the position of amino acids in their sequence as well as their physical structure, hydrophobicity, and molecular weight.

The obtained results could be referred to as the proteolytic action of *L. helveticus* on the hydrolysis of milk proteins, especially whey proteins and, in particular, α-Lactalbumin. In a similar vein, Griffiths and Tellez [54] showed that *L. helveticus* has a potent proteolytic system that can release amino acids from the casein matrix. Thus, it has a higher proteolytic activity than most other lactobacilli. The proteolytic system of *L. helveticus* consists of proteinases which initially cleave caseins into large peptides, peptidases, which further degrade these peptides to small peptides and amino acids, and specific transport proteins, which transport amino acids and peptides across the cytoplasmic membrane. The production of bioactive peptides from enzymatic hydrolysis of whey proteins has been studied. The opioid peptides, α-lactorphin and β-lactorphin, were released during in vitro proteolysis of whey proteins. *L. helveticus* was able to hydrolyze α-lactalbumin and release novel bioactive peptides with immunomodulatory properties [54,55]. Depending on the results obtained, the whey protein-based media is recommended to be used as a fermentation substrate for *L. helveticus*, especially when the follow-on formula is a matter of concern.

## 4. Conclusions

The prepared hydrolyzed follow-on formulas based on whey from camel and goat milks and containing fat blends closer to the composition of human milk fat in a postbiotic system increased the in vitro digestibility and improved antioxidant properties. So, it is highly recommended to use lactic acid proteolytic stains (e.g., *L. helveticus*) for the fermentation of follow-on formulas based on whey from camel or goat milks instead of formulas based on cow milk proteins. However, more in vitro and in vivo studies are needed to evaluate the beneficial effect of such formulas on infants’ health for long-term consumption. Thus, further investigation will focus on the in vivo bioavailability of follow-on formulas based on extracted whey from camel or goat milks compared to regular ones.

## Figures and Tables

**Table 2:** Chemical composition (mean ±SD) of prepared whey from different milk types.

Whey Type	Moisture %	TS %	Protein %	Fat %	Ash %	Lactose %
Cow milk-whey	92.85 ± 0.11 ^a^	7.15 ± 0.11 ^a^	2.00 ± 0.00 ^a^	0.50 ± 0.00 ^c^	0.79 ± 0.01 ^b^	3.87 ± 0.11 ^a^
Camel milk-whey	92.22 ± 0.24 ^a^	7.79 ± 0.24 ^a^	1.15 ± 0.06 ^c^	2.27 ± 0.13 ^a^	0.87 ± 0.01 ^a^	3.83 ± 0.11 ^a^
Goat milk-whey	93.59 ± 0.80 ^a^	6.41 ± 0.80 ^a^	1.43 ± 0.06 ^b^	1.20 ± 0.06 ^b^	0.67 ± 0.00 ^c^	3.11 ± 0.79 ^a^

^a–c^: There is no significant difference (*p* > 0.05) between any two means within the same column that have the same superscripted letters.

**Table 3:** Proteolysis degree (mmol tyrosine equivalent mL^−1^) of whey prepared from different milk types during fermentation by *L. helveticus* using the OPA method.

Whey Type	Fermentation Time (h)						
	Before Fermentation			During Fermentation			
	Z_1_	Z_2_	Z_3_	2 h	4 h	6 h	12 h
Cow milk-whey	2.47 ± 0.06 ^aE^	3.51 ± 0.25 ^aD^	3.41 ± 0.06 ^aD^	3.98 ± 0.06 ^aC^	4.90 ± 0.11 ^aB^	4.88 ± 0.14 ^bB^	6.15 ± 0.04 ^aA^
Camel milk-whey	1.64 ± 0.04 ^bB^	1.57 ± 0.08 ^cB^	1.57 ± 0.07 ^cB^	1.59 ± 0.14 ^bB^	1.67 ± 0.01 ^cB^	1.77 ± 0.05 ^cB^	2.17 ± 0.17 ^cA^
Goat milk -whey	1.17 ± 0.15 ^cE^	2.89 ± 0.12 ^bD^	3.08 ± 0.42 ^bD^	3.92 ± 0.09 ^aC^	3.75 ± 0.30 ^bC^	5.06 ± 0.12 ^aB^	5.74 ± 0.15 ^bA^

Z_1_: Fresh whey, Z_2_: Whey immediately after pasteurization, Z_3_: after *L. helveticus* inoculation, ^a–c^: There is no significant difference (*p* > 0.05) between any two means within the same column that have the same superscript letter. ^A–E^: There is no significant difference (*p* > 0.05) between any two means within the same row that have the same superscripted letters.

**Table 4:** DPPH radical scavenging activity (μmol Trolox equivalent mL^−1^) of whey prepared from different milk types during fermentation by *L. helveticus*.

Whey Type	Fermentation Time (h)					
	Before Fermentation		During Fermentation			
	Z_1_	Z_2_	2 h	4 h	6 h	12 h
Cow milk-whey	1.04 ± 0.12 ^aABC^	0.92 ± 0.02 ^aC^	1.01 ± 0.13 ^aBC^	1.08 ± 0.04 ^aAB^	1.17 ± 0.07 ^aA^	1.17 ± 0.03 ^aA^
Camel milk-whey	0.94 ± 0.19 ^bAB^	0.86 ± 0.14 ^aB^	0.97 ± 0.00 ^aAB^	0.97 ± 0.03 ^bAB^	0.97 ± 0.00 ^bAB^	1.01 ± 0.07 ^bA^
Goat milk-whey	0.88 ± 0.04 ^bAB^	0.76 ± 0.01 ^bB^	0.84 ± 0.01 ^bAB^	0.89 ± 0.02 ^bAB^	0.92 ± 0.06 ^bA^	0.95 ± 0.06 ^bA^

Z_1_: Fresh whey, Z_2_: Whey immediately after pasteurization. ^a,b^: There is no significant difference (*p* > 0.05) between any two means, within the same column have the same superscript letter. ^A–C^: There is no significant difference (*p* > 0.05) between any two means within the same row that have the same superscripted letters.

**Table 5:** Relative FAs (%) of different fats and oils used in the study.

Fatty Acids	Fat and Oil Type						
	Milk Fat Sources				Edible Vegetable Oils		
	Cow	Camel	Goat	Human	Rice Bran	Coconut	Canola
Saturated fatty acids							
Caprylic acid (C8:0)	3.05	-	2.92	1.19	-	15.35	-
Capric acid (C10:0)	7.16	-	13.71	7.91	-	15.27	-
Lauric acid (C12:0)	8.73	2.47	6.63	25.75	2.28	25.29	-
Myristic acid (C14:0)	18.75	16.92	12.10	12.57	3.10	28.25	0.43
Pentadecanoic acid (C15:0)	3.90	1.45	1.32	-	-	-	-
Palmitic acid (C16:0)	27.97	37.68	32.95	21.47	37.53	9.61	17.68
Stearic acid (C18:0)	3.92	6.10	5.79	3.22	2.84	1.41	2.52
Arachidic acid (C20:0)	-	-	-	-	0.79	-	0.8
Unsaturated fatty acids							
Palmitoleic (Cis-9, C16:1)	1.05	10.17	0.79	3.06	-	-	0.98
Oleic acid (C18:1n-9c)	11.88	25.21	17.47	24.84	53.47	4.82	74.82
Octadecanoic acid (cis-9,10, C18:2)	-	-	-	-	-	-	0.8
Gadoleic acid (cis-11, C20:1)	-	-	-	-	-	-	1.97
∑ SFAs	73.48	64.62	75.42	72.11	46.54	95.18	20.24
∑USFAs	12.93	35.38	18.26	27.90	53.47	4.82	78.57

**Table 6:** Formulation of fat blends based on different MF types.

Different Fat Blends	Fat and Oil (g 100 g^−1^)					
	Cow MF	Camel MF	Goat MF	Rice Bran Oil	Coconut Oil	Canola Oil
Cow MF-based	45	-	-	30	5	20
Camel MF-based	-	55	-	25	5	15
Goat MF-based	-	-	45	30	5	20

**Table 7:** Relative FA (%) distribution of recombined fat blends.

Fatty Acid	Different Fat Blends		
	Cow MF-Based	Camel MF-Based	Goat MF-Based
Saturated fatty acids			
Butyric acid (C4:0)	1.394	-	0.973
Caproic acid (C6:0)	0.766	-	1.027
Caprylic acid (C8:0)	1.013	0.705	1.734
Capric acid (C10:0)	1.339	0.586	4.233
Undecanoic acid (C11:0)	-	4.286	-
Lauric acid (C12:0)	4.621	-	5.883
Tridecanoic acid (C13:0)	0.030	0.024	-
Myristic acid (C14:0)	5.129	6.256	5.583
Pentadecanoic acid (C15:0)	0.529	0.374	0.389
Palmitic acid (C16:0)	18.779	20.803	18.062
Margaric acid (C17:0)	0.321	0.244	0.227
Stearic acid (C18:0)	4.028	5.195	3.713
Arachidic acid (C20:0)	0.053	-	-
Heneicosylic acid (C21:0)	0.025	-	-
Tricosylic acid (C23:0)	0.033	0.032	0.031
Unsaturated fatty acids			
Myristoleic acid (C14:1n5)	0.369	0.494	0.135
Pentadecanoic acid (C15:1n5c)	0.090	0.138	0.090
Palmitoleic acid (C16:1n7c)	0.622	3.533	0.344
Heptadecenoic acid (C17:1)	0.148	0.162	0.104
Elaidic acid (C18:1n-9t)	0.911	0.748	0.572
Oleic acid (C18:1n-9c)	36.648	34.526	34.467
Linolelaidic acid (C18:2n6t)	0.098	0.092	0.069
Linoleic acid (LA: cis-9,10, C18:2)	17.951	17.132	17.468
Linolenic acid (C18:3n3)	0.510	0.524	0.507
α-Linolenic acid (ALA: cis-,12,15 C18:3n3)	3.433	3.089	3.348
Cetoleic acid (C20:1n9)	0.535	0.527	0.544
Gadoleic acid (cis-11, C20:1)	-	-	0.014
Eicosadienoic acid (C20:2n6)	0.032	-	-
EicosatrienoicS acid (C20:3n3)	0.220	0.202	0.219
Eicosatetraenoic acid (EPA: C20:5n3)	0.220	0.214	0.204
Erucic acid (C22:1n9c)	-	0.066	-
Docosadienoic acid (C22:2n6)	0.026	-	-
Nervonic acid (C24:1n9)	0.047	0.046	0.055
∑ SFAs	38.148	38.508	41.860
∑ MUSFAs	38.459	39.493	53.739
∑ PUSFAs	22.385	21.161	21.760
∑ Trans FAs	1.008	0.840	0.641

**Table 8:** Added components (g 100 mL^−1^ whey) to prepare different whey samples to reach the required percentage of each component in the different follow-on formulas.

Different Based Follow-On Formula	Component (g 100 mL^−1^)									
	Required Protein (2.9% or 1.9%) ^a^		Required Fat (4%)						Required Lactose (7%)	Lecithin (0.5%)
			Milk Fat Source			Vegetable Edible Oil				
	Casein Powder	Freeze Dried Whey	Cow MF	Camel MF	Goat MF	Rice Bran Oil	Coconut Oil	Canola Oil		
Follow-on formula 1 ^▪^	0.9	-	1.3	-	-	1.2	0.2	0.8	3.7	0.5
Follow-on formula 2 ^▪▪^	0.6	0.3	-	-	-	1.0	0.2	0.6	3.4	0.5
Follow-on formula 3 ^▪▪▪^	0.6	-	-	-	0.6	1.2	0.2	0.8	3.9	0.5

^a^: the percent of total protein was 2.9% for cow whey-based follow-on formula and 1.9% for both camel whey-based follow-on formula and goat whey-based follow-on formula. ^▪^ Average of cow milk whey composition used in formula 1 (2% protein, 0.5% fat, and 4% lactose). ^▪▪^Average of camel milk whey composition used in formula 2 (1.13% protein, 2.2% fat, and 3.59% lactose). ^▪▪▪^Average of goat milk whey composition used in formula 3 (1.44% protein, 1.20% fat, and 3.11% lactose).

**Table 9:** Effect of in vitro digestibility on hydrolysis degree and antioxidant activity of different follow-on formulas against commercial follow-on formulas and human milk.

Follow-On Formula and Human Milk	Total Free Amino Acids (mmol Tyrosine Equivalent mL^−1^)		DPPH Radical Scavenging Activity (μmol Trolox Equivalent mL^−1^)		Reducing Power (μg Ascorbic Acid Equivalent mL^−1^)	
	Before and after In Vitro Digestion					
	Before Digestion	After Digestion	Before Digestion	After Digestion	Before Digestion	After Digestion
Non-fermented follow-on formula						
Cow whey-based: (2.9% protein)	0.80 ± 0.0 1 ^bB^	1.07 ± 0.08 ^cA^	3.90 ± 0.14 ^cdB^	20.58 ± 0.12 ^eB^	8.30 ± 0.24 ^eB^	12.66 ± 0.23 ^fA^
Camel whey-based: (2% protein)	0.71 ± 0.16 ^bcB^	1.33 ± 0.05 ^bA^	4.25 ± 0.12 ^bcB^	21.09 ± 0.2 1b ^cA^	7.63 ± 0.12 ^fB^	9.84 ± 0.12 ^bA^
Goat whey-based: (2% protein)	0.54 ± 0.05 ^cdA^	0.58 ± 0.05 ^dA^	5.49 ± 0.09 ^aB^	20.70 ± 0.12 ^cA^	9.84 ± 0.12 ^cB^	19.09 ± 0.12 ^dA^
fermented follow-on formula						
Cow whey-based: (2.9% protein)	1.21 ± 0.02 ^aB^	1.35 ± 0.14 ^bA^	4.62 ± 0.10 ^bB^	21.35 ± 0.29 ^abA^	8.63 ± 0.31 ^dB^	11.98 ± 0.13 ^gA^
Camel whey-based: (2% protein)	1.14 ± 0.06 ^aB^	1.33 ± 0.14 ^bA^	4.27 ± 0.10 ^bcB^	21.04 ± 0.25 ^bcA^	8.83 ± 0.12 ^dB^	9.91 ± 0.35 ^hA^
oat whey-based: (2% protein)	0.84 ± 0.13 ^bA^	0.94 ± 0.12 ^cA^	5.70 ± 0.06 ^aB^	21.47 ± 0.28 ^abA^	11.18 ± 0.24 ^bB^	20.10 ± 0.23 ^cA^
Commercial follow-on formula						
Commercial formula 1	0.50 ± 0.07 ^cdB^	0.93 ± 0.11 cA	1.47 ± 0.27 ^eB^	21.81 ± 0.31 ^aA^	4.94 ± 0.18 ^gB^	20.97 ± 0.14 ^bA^
Commercial formula 2	0.17 ± 0.02 ^eB^	1.86 ± 0.11 ^aA^	4.57 ± 0.13 ^bB^	21.65 ± 0.37 ^aA^	7.11 ± 0.39 ^gB^	17.89 ± 0.12 ^eA^
Human milk	0.40 ± 0.20 ^deB^	0.58 ± 0.27 ^dA^	3.43 ± 0.20 ^dB^	20.75 ± 0.78 ^cA^	18.02 ± 0.25 ^aB^	22.58 ± 0.18 ^aA^

^a–h^: There is no significant difference (*p* > 0.05) in each parameter between any two means within the same column that have the same superscript letter; ^A–B^: There is no significant difference (*p* > 0.05) in each parameter between any two means within the same row that have the same superscripted letters.

## Data Availability

Data are contained within the article.
